# Impulsivity and Stress Response in Pathological Gamblers During the Trier Social Stress Test

**DOI:** 10.1007/s10899-017-9685-3

**Published:** 2017-03-18

**Authors:** G. Maniaci, A. E. Goudriaan, C. Cannizzaro, R. J. van Holst

**Affiliations:** 1 Department of Experimental Biomedicine and Clinical Neurosciences, University of Palermo, Palermo, Italy; 2 Academic Medical Center, Department of Psychiatry, Amsterdam Institute for Addiction Research, University of Amsterdam, Amsterdam, The Netherlands; 3 Arkin Mental Health Care, Amsterdam, The Netherlands; 4 Department of Sciences for Health Promotion and Mother and Child Care “Giuseppe D’Alessandro”, University of Palermo, Palermo, Italy

**Keywords:** Gambling disorder, Pathological gambling, Problem gambling, Stress response, Impulsivity, Trier Social Stress Test, Cortisol, Heart rate

## Abstract

Gambling has been associated with increased sympathetic nervous system output and stimulation of the hypothalamic–pituitary–adrenal axis. However it is unclear how these systems are affected in pathological gambling. This study aimed to investigate the effect of the Trier Social Stress Test (TSST) on cortisol and on cardiac interbeat intervals in relation to impulsivity, in a sample of male pathological gamblers compared to healthy controls. In addition, we investigated the correlation between the TSST, duration of the disorder and impulsivity. A total of 35 pathological gamblers and 30 healthy controls, ranging from 19 to 58 years old and all male, participated in this study. Stress response was measured during and after the TSST by salivary cortisol and cardiac interbeat intervals; impulsivity was assessed with the Barratt Impulsiveness Scale (BIS-11). Exposure to the TSST produced a significant increase in salivary cortisol and interbeat intervals in both groups, without differences between groups. We found a negative correlation between baseline cortisol and duration of pathological gambling indicating that the longer the duration of the disorder the lower the baseline cortisol levels. Additionally, we found a main effect of impulsivity across groups on interbeat interval during the TSST, indicating an association between impulsivity and the intensity of the neurovegetative stress response during the TSST. Involvement of the hypothalamic–pituitary–adrenal axis in pathological gambling was confirmed together with evidence of a correlation between length of the disorder and diminished baseline cortisol levels. Impulsivity emerged as a personality trait expressed by pathological gamblers; however the neurovegetative response to the TSST, although associated with impulsivity, appeared to be independent of the presence of pathological gambling.

## Introduction

In the last few years a substantial body of scientific literature has highlighted common elements regarding clinical and neurobiological mechanisms of pathological gambling and substance use disorders (Fauth-Buhler et al. 2016; Leeman and Potenza 2012; Petry et al. 2013; Goudriaan et al. 2014). Indeed, activation of the dopaminergic mesolimbic system represents the neurobiological substrate of the reinforcing properties and of the enhanced incentive salience both of drugs of abuse and addictive behaviours like pathological gambling (Brancato et al. 2014; Berridge 2007). Thus, in the new edition of the Diagnostic and Statistical Manual of Mental Disorders (DSM-5) pathological gambling has been identified as a behavioural addictive disorder and moved to the category “Subtance-Related and Addictive Disorders” (American Psychiatric Association. Diagnostic and statistical manual of mental disorders—5th ed. 2013). In The Netherlands, pathological gambling prevalence ranges approximately from .25 to .75%, similar to rates in several other European countries and often is associated to comorbidity with other psychiatric disorders thus provoking negative psychosocial consequences and health costs (Cowlishaw et al. 2012; Griffiths 2009; Goudriaan 2007; Lejoyeux 2012; Maniaci et al. 2015; Odlaug et al. 2013).

From clinical and neuropsychological studies it becomes clear that impulsivity is an important factor that contributes to the onset and worsening of pathological gambling (Blanco et al. 1996; Steel and Blaszczynski 1998; Van Holst et al. 2010; Verdejo-Garcia et al. 2008). Impulsivity consists in the predisposition toward rapid, unplanned reactions to internal or external stimuli with no regard to their negative consequences, and the occurrence of impulsivity leads subjects to be highly responsive to immediate positive reinforcement but rather insensitive to long-term negative consequences (Moeller et al. 2001). Impulsivity is related to impaired decision-making and higher risk behaviour in pathological gamblers (PGs; Alessi and Petry 2003; Cavedini et al. 2002; Goudriaan et al. 2005; Petry and Casarella 1999; Zermatten et al. 2005). Compared to healthy controls (HCs), PGs display increased levels of impulsivity (Marazziti et al. 2014; for a review see Goudriaan et al. 2014) and, notably score significantly higher on impulsivity and on the inability to resist craving compared to alcoholics and cocaine users (Grant et al. 2016). Moreover the presence of high impulsivity levels is considered an important factor for treatment failure in pathological gambling (Leblond et al. 2003; Goudriaan et al. 2008). The physiological and systemic responses correlated with impulsivity include a variety of signs such as heart rate and electrodermal activity (EDA) (Derefinko et al. 2014; Mathias and Stanford 2003). For instance, high impulsive and low impulsive subjects show different patterns of psychophysiological reactivity in a gambling task during an active choice and high bet size; indeed differences in heart rate accelerations to wins versus losses are positively correlated with impulsivity levels in healthy students (Studer and Clark 2011; Studer et al. 2016). Furthermore higher impulsivity scores are associated with reduced electrodermal activity (EDA) differences between wins and losses and reduced EDA in response to stress during a risky choice task (Stankovic et al. 2014). However, how impulsivity is associated with stress responses in pathological gambling is not yet clear (Kräplin et al. 2014).

Several studies have observed increased physiological arousal in recreational gamblers while engaging in gambling related activities, such as heart rate and hypothalamic–pituitary–adrenal axis (HPA) activation (Coventry and Constable 1999; Kreuger et al. 2005; Wulfert et al. 2008). Gambling has been shown to lead to moderate heart rate elevation, and to increased levels of salivary cortisol (Meyer et al. 2000). Unexpectedly, problem gamblers did not show significant differences in plasma cortisol levels during casino gambling, compared to HCs (Meyer et al. 2004). Rather, salivary cortisol levels before and after watching gambling scenarios were significantly higher in recreational gamblers compared to PGs. These findings suggest that pathological gambling is associated with hyporeactivity of the stress system to cue-related stimuli (Paris et al. 2009). In this regard lower concentrations of salivary cortisol are associated with riskier choices and monetary losses in the Iowa Gambling Task, whereas the opposite pattern—higher concentrations of salivary cortisol—is associated with less risky choices and monetary gain (van Honk et al. 2003). However, whether lower HPA axis responses to cue-related stimuli are associated with a longer duration and intensity of pathological gambling is still not clear. In addition, it is unknown whether HPA-axis reactivity in PGs is diminished only in gambling situations, or whether a more general dysregulation of the HPA-axis reactivity exists. A dysfunctional arousal of the HPA axis could be related to a more severe form of pathological gambling or longer presence of pathological gambling, since diminished HPA-axis reactivity could maintain the addictive behaviour, because risky behavior or losses do not activate the HPA-axis enough, and thus reinforce the inclination towards risky behaviour or continuation of gambling, despite losses.

For these reasons the present study investigated HPA-axis reactivity to a psychosocial stress in PGs compared to HCs using the Trier Social Stress Test (TSST). This test has been shown to lead to a robust increase in cortisol through the activation of HPA axis and sympathetic nervous system (Brkic et al. 2015; Dickerson and Kemeny 2004; Inagaki and Eisemberger 2015; Kirschbaum and Hellhammer 1994). We hypothesised that relative to HCs, PGs would display a different physiological response to the stress test. Moreover considering that hypocortisolism may be a consequence of exposure to chronic stress (Heim et al. 2000), we also investigated the potential occurrence of a negative correlation between the intensity of the physiological stress response and duration of pathological gambling (Fernald et al. 2008). Because higher impulsivity levels have been correlated with increased sympathetic nervous system activity (Kreuger et al. 2005) and decreased cardiac vagal control in male adolescents (Allen et al. 2000) we wanted to investigate the interaction between impulsivity and stress reactivity in PGs, as higher impulsivity has been frequently reported in PGs. Impulsive psychological traits may correlate with the stress response of PGs during psychosocial stressing situations such as TSST, and therefore we investigated if there was a different pattern of correlation between impulsivity and stress response in PGs compared to HCs.

## Materials and Methods

### Participants

A total of 35 PGs and 30 HCs, ranging from 19 to 58 years old, participated in this study. PGs were recruited from a local addiction treatment center where they received cognitive behavioural therapy for pathological gambling. HCs were recruited through advertisements in local newspapers. Because most treatment-seeking PGs are men, only male participants were included in the study. Exclusion criteria for both groups, evaluated through the Composite International Diagnostic Interview (CIDI), were: lifetime diagnosis of schizophrenia or psychotic episodes; diagnosis of manic disorder (CIDI, section F), obsessive compulsive disorder (CIDI, section E), alcohol use disorders (CIDI, section J), substance dependent disorder (CIDI, section L) or post-traumatic stress disorder (CIDI, section K); treatment for mental disorders other than pathological gambling in the past 12 months; use of psychotropic medication; difficulty reading Dutch; IQ below 80 (measured by the Dutch Adult Reading Test; Schmand et al. 1992); age under 18 years old; positive urine screen for alcohol, amphetamines, benzodiazepines, opioids or cocaine; history or current treatment for neurological disorders, major internal disorders, brain trauma, or exposure to neurotoxic factors. In addition, HCs were excluded if they gambled more than twice a year. The study was approved by the ethical review board of the Academic Medical Center in Amsterdam, the Netherlands, all participants gave written informed consent and all measures were administered under respect of privacy. This study was part of a larger research project including MRI examination (van Holst et al. 2012a, b, c) and behavioural experiments (Starcke et al. 2013). Participants were reimbursed with 50 Euros transferred to their bank account following participation.

### Measure of Stress Induction

A modified version of the TSST was used as psychosocial stressor. Stress is an important factor known to increase alcohol and drug relapse risk (Sinha 2007). The TSST is one of the most reliable and standardized tests for studying stress responses in different types of individuals and personalities (Dickerson and Kemeny 2004; Inagaki and Eisemberger 2015; Kirschbaum et al. 1993a, b). The TSST is a psychosocial stress test and therefore closely resembles frequently encountered stressors in real life. It was shown that the TSST induced increased craving for alcohol in moderate-to-heavy social drinkers (Nesic and Duka 2008). Moreover psychosocial stress is a relevant condition that can push an individual to search for stress relief by engaging in gambling, and therefore, studying psychosocial stress has relevance for pathological gambling.

In the TSST, participants have to perform two different tasks in front of a selection committee and a video camera. The committee consists of three experimenters introduced as being trained in “behavioural observation”. Participants were welcomed and informed about the research project by the test instructor and were asked to start filling in personality questionnaires. The start of the TSST was signaled by the exit of the familiar test instructor and the entrance of a ‘psychologist’, who introduced her/himself to the participant and gave the instruction to prepare a speech for a job interview for one of their favourite jobs. Participants are told that their performance is recorded on video to later analyze voice pitch and nonverbal behaviour and are asked to face a one-way mirror screen when giving the speech so that experts behind the mirror could judge them. The participants got 10 min to prepare the speech and the speech had to be no longer than 5 min. After the participant finished the speech, he or she had to do an arithmetic task (participants were asked to make subtractions of 7 from 1029, and in case no errors were made during the first 5 subtractions, a switch was made to subtractions of 13). The psychologist did not provide any further feedback and acted in a very cold and reserved manner. The recovery period was set to consist of 45 min after completion of the arithmetic task.

### Measures of Stress Response

#### HPA Axis Response

Cortisol was collected six times by using Kirschbaum’s protocol (Kirschbaum et al. 1993b) (see Table 1). Cortisol levels were determined from saliva samples representing the unbound biologically active hormone fraction. Salivary cortisol is highly correlated with serum free and total cortisol levels and has been shown to be independent of saliva flow rate. For easy and hygienic sampling of saliva, the Salivette sampling device (Sarstedt, Nümbrecht, Germany) was employed. Participants chewed on each salivette for 1 min for each measurement. Data from the last measurement was lost for two PGs and four HCs. Samples were stored at −20 °C until being assayed. Saliva samples were sent to the laboratory of the University of Dresden (Germany; laboratory of prof. Kirschbaum) to determine cortisol levels. Free cortisol levels were measured using a commercial chemiluminescence immunoassay (IBL International, Hamburg, Germany).

**Table 1:** Procedure of salivary cortisol measurement

Salivary cortisol measurements	TSST steps	Time (minutes)
T1	Baseline	−20
T2	Start preparation	0
T3	Start speaking task	10
T4	End cognitive task	18
T5	20 min after end stress task	40
T6	40 min after end stress task	60

#### Sympathetic Nervous System Response

Throughout the TSST, an electrocardiogram (ECG) signal was recorded continuously to monitor heart rate variability. The Vrije Universiteit- Ambulant Monitoring System (AMS) 5 fs-SCL version (Skin Conductance Level) was connected 30 min prior to initiation of the stress task. Seven active Ag/AgCL electrodes (10 mm, Ultra trace) were used. The first electrode was placed slightly below the collar bone, 4 cm to the right of the sternum. The second one was placed between the lower two ribs, just right of the sternum. The third electrode was placed at the jugular notch, just above the sternum, while the fourth electrode was placed at the xiphoid process, just under the sternum, both in the medial line. Finally the sixth and seventh electrodes were placed dorsally, on the spine, 3 cm above electrode 4 and 3 cm below electrode five respectively. The ECG signal was led into a differential amplifier with an input impedance higher than 1 MO. The amplified ECG was then passed through a band pass filter at 17 HZ after which it was used for R-peak triggering. At each R-peak, a millisecond counter was read and reset, yielding inter beat interval (IBI) (see Table 2). Data from 29 PGs and 23 HCs were analysed for all sessions, except for the last IBI session 40 min after the end of the stress task, there because of technical failure only 15 PGs and 23 HCs were included.

**Table 2:** Procedure of IBI interval extraction

IBI interval extraction	TSST steps	Time (minutes)
T1	Baseline	−20
T2	Start preparation	0–2
T3	Start speaking task	10–12
T4	End cognitive task	18–20
T5	20 min after end stress task	40–42
T6	40 min after end stress task	60–62

### Psychological Measures

#### Gambling Behaviour Assessment

All participants completed the South Oaks Gambling Screen (SOGS). The SOGS is a 20 items questionnaire that measures problematic gambling behaviour through questions on participant’s history of gambling, the frequency at which the person engages in these behaviours, and obstacles that gambling may have posed in the participant’s life. The total score on the SOGS ranges from 0 to 20, scores from 5 indicate probable pathological gambling (Lesieur and Blume 1987).

#### Gambling Diagnosis and Exclusion Criteria

To assess the diagnostic criteria for a DSM-IV-TR diagnosis and to evaluate the exclusion criteria of this study, the Composite International Diagnostic Interview (CIDI) was used. The CIDI is a comprehensive standardized diagnostic interview designed for assessing mental disorders according to the DSM-IV (WHO 1997).

#### Impulsivity

To evaluate impulsiveness we used the Barratt Impulsiveness Scale, 11th version (BIS-11; Barratt 1985). The BIS-11 is a self-report questionnaire, which contains 30 questions that need to be scored on a scale from 1 to 4 (1 = rarely/never; 2 = occasionally; 3 = often; 4 = almost always/always). Factor analysis includes 6 first order factors (attention, motor impulsiveness, self-control, cognitive complexity, perseverance and cognitive instability) and three second order factors (attentional impulsiveness, motor impulsiveness and non-planning impulsiveness). The BIS-11 total score indicates the level of impulsiveness. The higher the BIS-11 total score, the higher the impulsiveness level is. The questionnaire contains statements that indicate impulsive behaviour (‘I do things without thinking’) and statements that indicate non-impulsive behaviour (‘I am self-controlled’). The BIS-11 is the most frequently used self-report measure of impulsivity.

### Procedure

After the informed consent was signed, participants filled out questionnaires. Then they started the modified version of the TSST described above. During the TSST salivary cortisol and heart rate variability were collected. All testing sessions took place between 1 and 4 PM to ensure that there were no large variations in cortisol secretion between participants due to circadian rhythm (Kudielka et al. 2004).

## Statistical Analyses

One-way analysis of variance (ANOVA) was used to evaluate significant differences between PGs and HCs in age, BIS-11 and SOGS scores. A repeated measures ANOVA with “time” as within-subjects factor and “group” as between-subjects factor for both collected stress measures (salivary cortisol and IBI) was performed. Greenhouse-Geisser corrected p-values were used when appropriate. Partial eta-squared (*ηp*2) was used as a measure of effect size. Bivariate Pearson’s correlation was used to verify a correlation between stress measures and the duration of the disorder. To explore the relationship between impulsivity, gambling behaviour and the physiological stress measures during the TSST, a mixed model analysis was used. All analyses were performed with an alpha of .05. All statistical analyses were conducted using SPSS for Windows 22.0.

## Results

### Sample Characteristics and Differences in Impulsiveness

Table 3 includes the descriptive data of both samples; there were no significant differences between groups in age. The groups were significantly differed on impulsivity (F = 4.92; *p* = .042) and gambling behaviour scores (F = 280.18; *p* < .001). The duration of the pathological gambling had a mean of 8.51 years (SD = 10.18).

**Table 3:** One-way ANOVA between groups by age, South Oaks Gambling Screen (SOGS) and Barratt Impulsiveness Scale—11 version (BIS-11) scores

Factor	Pathological gamblers (n = 35) mean (SD)	Healthy controls (n = 30) mean (SD)	Test One-way ANOVA F
AGE	36.34 (10.871)	37.60 (10.294)	.227 (.636)
SOGS	10.62 (2.96)	.43 (1.65)	280.18****
BIS-11	54.82 (7.09)	51.13 (7.25)	4.291*

* *p* < .05; ** *p* < .01; *** *p* < .005; **** *p* < .001

*SOGS* South Oaks Gambling Screen

*BIS-11* Barratt Impulsiveness Scale—11 version

### Stress Reactivity During and After the TSST

**Fig. 1:**
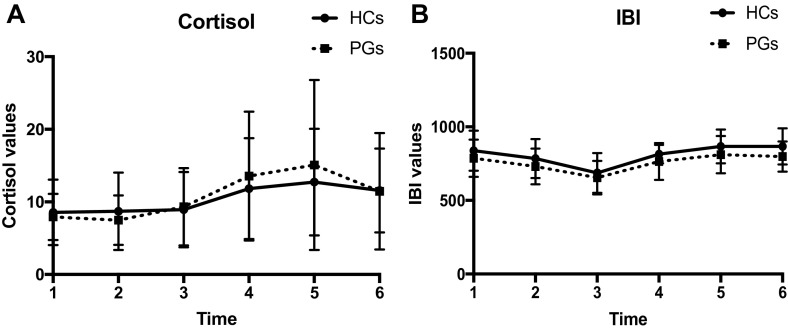
Cortisol (**a**) and Interbeat Interval (IBI) (**b**) levels at baseline, during stress induction, and after cessation of the TSST in PGs and HCs

A significant main effect of time was found with two separate repeated measures ANOVA, meaning that the TSST significantly activated the HPA axis through an increase during the TSST (and subsequent decrease) of salivary cortisol [F(1, 57) = 18.332; *p* = .000001] and the sympathetic nervous system through a modification of the IBI [F(1, 35) = 59.652; *p* = .000000] in both groups. However we found no significant difference between groups, nor interaction between group * time in both parameters during or after the TSST (Table 4). Although we didn’t find significant differences between groups on cortisol levels, at T4 and T5 a trend was visible for higher cortisol levels in PGs compared to HCs (Fig. 1).

**Table 4:** Repeated measures ANOVA for cortisol and IBI

Factor	F	df	MSE	*p*	*ηp*2
Cortisol: time	18.332	1,57	1158.42	****	.243
Cortisol: group	.024	1,57	4.568	.878	4.568
Cortisol: time × group	1.233	1,57	77.92	.291	.024
IBI: time	59.652	1,35	247,600.35	****	.630
IBI: group	3.756	1,35	286,270.35	.060	.094
IBI: time × group	.388	1,35	1610.18	.815	.011

* *p* < .05; ** *p* < .01; *** *p* < .005; **** *p* < .001

### Stress Response and “Duration” of the Disorder

The association between the duration of pathological gambling and the physiological stress response to the TSST was explored using a bivariate Pearson’s correlation. Consistently with our hypothesis we found a negative correlation between the duration of pathological gambling and cortisol at baseline (T1) (r = −.493; *p* = .003) and also at T2 (r = −.355; *p* = .036). Thus, increased duration in pathological gambling problems correlates to lower cortisol levels (Table 5). We found no significant correlations between the duration of the disorder and sympathetic nervous system activation during the TSST (Table 6).

**Table 5:** Bivariate Pearson’s correlation between “duration” of the disorder and salivary cortisol

Factor	T1	T2	T3	T4	T5	T6
Duration	−.493**	−.355*	−.296 (.085)	−.313 (.067)	−.286 (.096)	−.264 (.138)

* *p* < .05; ** *p* < .01; *** *p* < .005; **** *p* < .001

**Table 6:** Bivariate Pearson’s correlation between “duration” of the disorder and IBI

Factor	T1	T2	T3	T4	T5	T6
Duration	−.102 (.599)	−.029 (.883)	−.113 (.560)	−.113 (.558)	−.144 (.455)	−.119 (.588)

* *p* < .05; ** *p* < .01; *** *p* < .005; **** *p* < .001

### Impulsivity and Stress Response

The relationship between impulsivity and stress response during the TSST in PGs and in HCs was investigated through a mixed model analysis. There was a significant main effect of impulsivity on IBI during the stress test [F(1, 48) = 12.512, *p* = .001] independently from a group effect (Table 7).

**Table 7:** Mixed model analysis for impulsivity, cortisol and IBI

Factor	F	df	*p*
Impulsivity: cortisol	14.998	1,61	.090
Impulsivity: group	.524	1,61	.472
Impulsivity: cortisol × group	.795	1,61	.415
Impulsivity: IBI	12.512	1,48	***
Impulsivity: group	1.271	1,48	.265
Impulsivity: IBI × group	.269	1,48	.443

* *p* < .05; ** *p* < .01; *** *p* < .005; **** *p* < .001

## Discussion

This study investigated the effects of psychological stress on HPA axis activation and sympathetic nervous system response in relation to impulsivity in a sample of male PGs, compared to HCs. In particular, we measured salivary cortisol levels and IBI in PGs during and after the TSST compared to the HC response. The impact of the duration of the disorder and the role played by impulsivity in the onset of modifications in the physiological response to the stress test were also assessed.

Our study shows that the longer the duration of pathological gambling the lower the cortisol response before psychosocial stress, which is consistent with findings indicating that exposure to chronic stressful situations—similarly to gambling—can lead to hypocortisolism (Heim et al. 2000). However, there was no evidence of differences in IBI between groups. Several studies have highlighted an association between severity of psychiatric disorders and cortisol levels, such as in post-traumatic stress disorder (Yehuda et al. 1996), generalised anxiety disorder (Steudte et al. 2011) and pathological gambling (Geisel et al. 2015); consistently, our data report that a longer duration of pathological gambling is related to lower baseline salivary cortisol levels. This correlation can be interpreted as a maladaptive response of PGs’ HPA axis to the chronic distress caused by enduring gambling behaviour, thus confirming the occurrence of a dysregulation of critical central neuroendocrine circuits in pathological gambling. A theory suggests that under the influence of continuous or intermittent chronic stressful stimulus the hypocortisolism might be an adaptive self-preserving response, in order to protect the metabolic machinery, and most importantly, the brain (Hellhammer and Wade 1993). States of hypocortisolism are common in patients chronically exposed to stressful environments, in those with unpredictable schedules and in those with traumatic early life experiences (Gunnar and Vazquez 2001; Heim et al. 2000). As in several stress related disorders, also in pathological gambling the repeated or intermittent distress, caused by enduring gambling behaviour, can produce an alteration of basal HPA axis activity finally leading to the observed hypocortisolism. Notably, in PGs the baseline hypocortisol condition might promote increased vulnerability for the development of physical consequences, such as high-stress sensitivity, chronic fatigue and chronic pain (Fries et al. 2005), leading to functional and anatomical disturbances that need medical intervention. However when performing the TSST, PGs, independently from the duration of the disorder, display a remarkable activation of the HPA axis. Indeed there was a trend for higher cortisol levels at T4 and T5 in PGs compared to HCs. This suggests a vulnerability of the stress system of PGs to environmental and social challenges. Future longitudinal studies should further investigate this issue and clarify whether the correlation between hypocorticolism and duration of the disease observed in this study is a cause or a consequence of pathological gambling.

Although we replicated the robust effect of the TSST to produce an increase of salivary cortisol and IBI, we did not find significant differences between groups. This finding suggests that in PGs as in HCs, neuroendocrine and neurovegetative responses to non gambling-related stress stimuli are not compromised, at least in our experimental conditions, and for the examined parameters.

Interestingly, we show that impulsivity is positively correlated with IBI, over and above the influence of pathological gambling. Indeed, higher impulsivity subjects revealed significantly higher IBIs throughout the TSST. Thus impulsivity, independently of pathological gambling, appears to be related to a decreased heart rate during the TSST, consistent with an earlier study on the relationship between impulsivity and cardiovascular responses during a psychosocial stress task (Allen et al. 2009). Thus, our findings imply that impulsivity—regardless of the presence of pathological gambling—is associated with a diminished cardiovascular responsivity. Impulsivity, particularly during young adult life, has been implicated in an increase in the risk of unhealthy behaviours and developments due to structural brain deficits and lack of experience with novel adult behaviours (Romer 2010). This study goes some way to indicate a potential biological mechanism associated with impulsivity. Central motivational dysregulation has been proposed to underpin the link between blunted stress reactivity and outcomes such as obesity, depression, a range of substance and behavioural dependencies, and bulimia (Carroll et al. 2009, 2011; Lovallo 2011). Thus, blunted stress reactivity can be considered a peripheral marker of dysregulation of the neural systems that support motivation, emotional regulation, and goal-directed behaviour. The association between impulsivity and dysregulation of the neurovegetative stress response deserves special attention, because it represents a vulnerability factor that can affect impulsive decision making (Brand et al. 2005; Goudriaan et al. 2005), thus exacerbating all those neuropsychiatric disorders characterized by impaired impulse control.

The main limitation of the present study is the recruitment of only male participants. Further studies should be carried out in order to investigate the relation between impulsivity and physiological stress response in PGs including females. However, by including only males suffering from pathological gambling, whithout other comorbid disorders, we made sure that we had a quite homogeneous group, limiting the potentional confounding factors in our study.

In conclusion, an influence of pathological gambling on HPA-axis activity was highlighted together with a role of impulsivity in the cardiac stress response to a psychological stress test. Future studies should test whether the stress response normalizes after treatment and whether a recovered neuro-humoral reactivity to stress is associated with diminished relapse.
